# Supplementary figures and images for: Short communication: Feasibility of dengue vaccine to infect different human cell lines: An alternative potency test using HEK293T cells
Source: PLoS One. 2022 May 6;17(5):e0267653. doi: 10.1371/journal.pone.0267653 (PMC9075668; doi:10.1371/journal.pone.0267653)

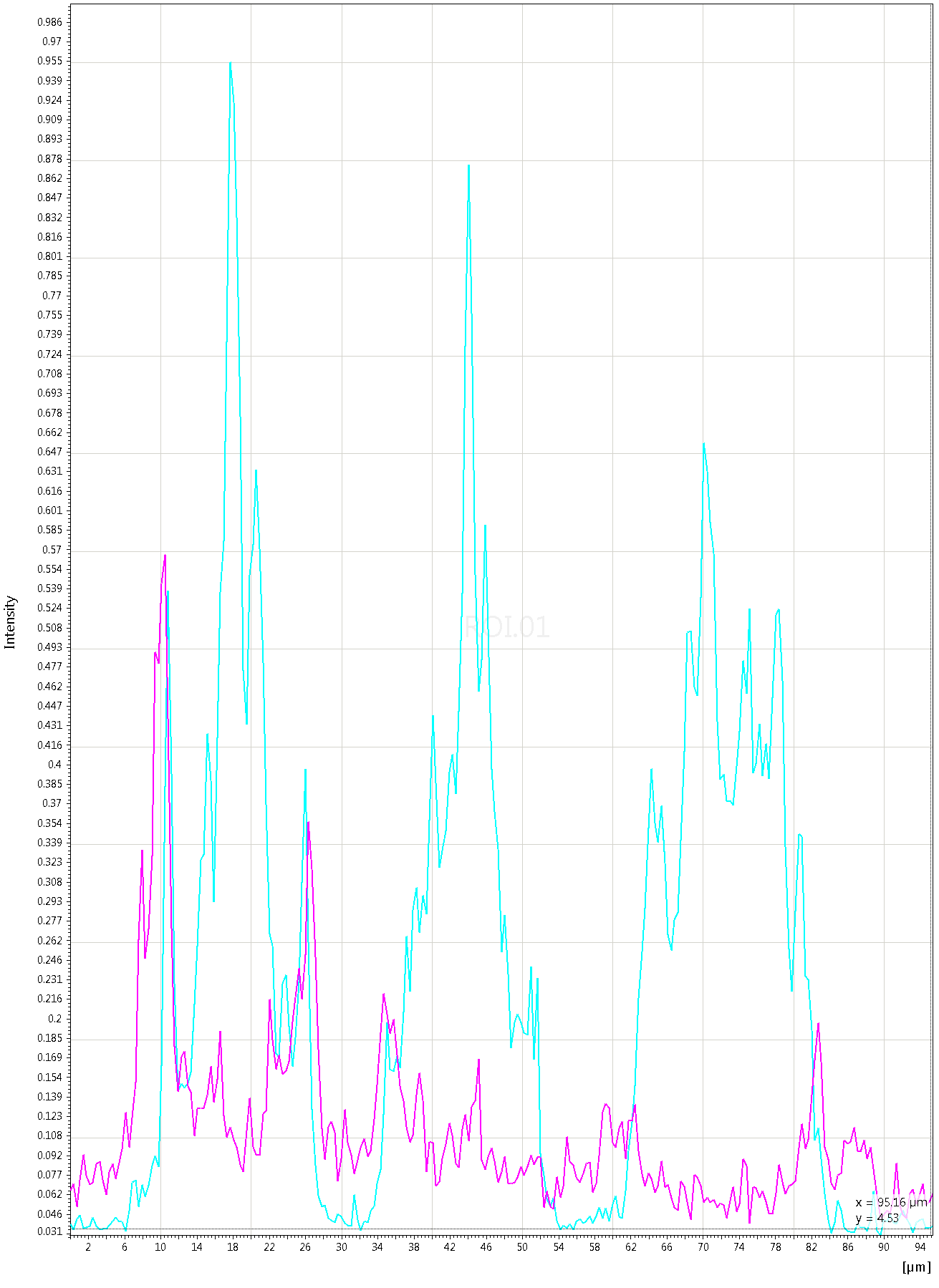

Supplement: S1 Fig — Cells were stained with anti-4G2 (pan-flavivirus antibody, pink fluorescence) and PAFI (DNA staining, blue fluorescence). Representative graph of fluorescence intensity (y-axis) against the distance in micrometer (x-axis) of infected VERO cells, obtained from confocal representative image (confocal microscope Leica TCS-SP8 at 200X magnification). n = 2. (TIF) [file pone.0267653.s001.tif]

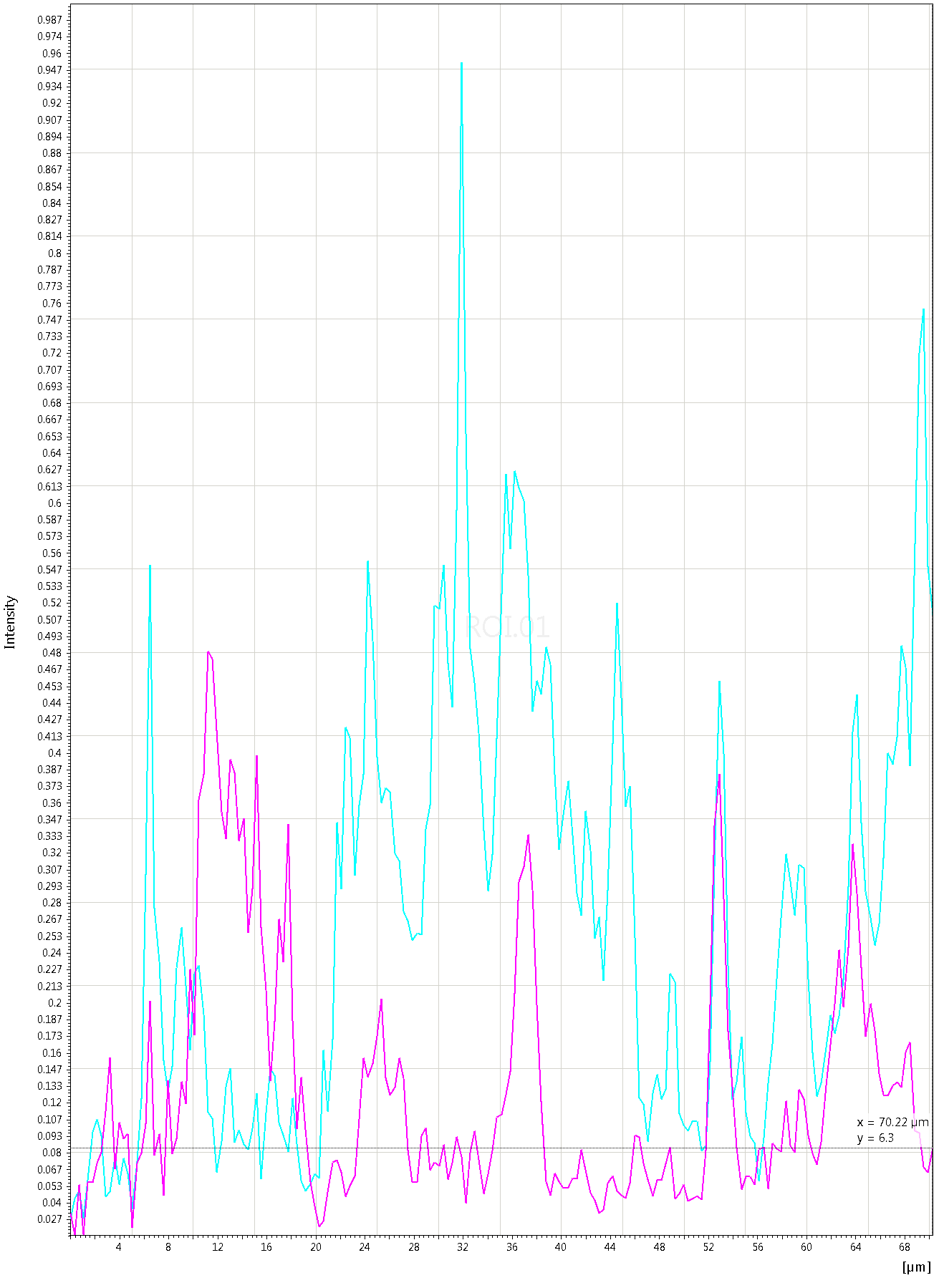

Supplement: S2 Fig — Cells were stained with anti-4G2 (pan-flavivirus antibody, pink fluorescence) and PAFI (DNA staining, blue fluorescence). Representative graph of fluorescence intensity (y-axis) against the distance in micrometer (x-axis) of infected HEK293T cells, obtained from confocal representative image (confocal microscope Leica TCS-SP8 at 200X magnification). n = 2. (TIF) [file pone.0267653.s002.tif]

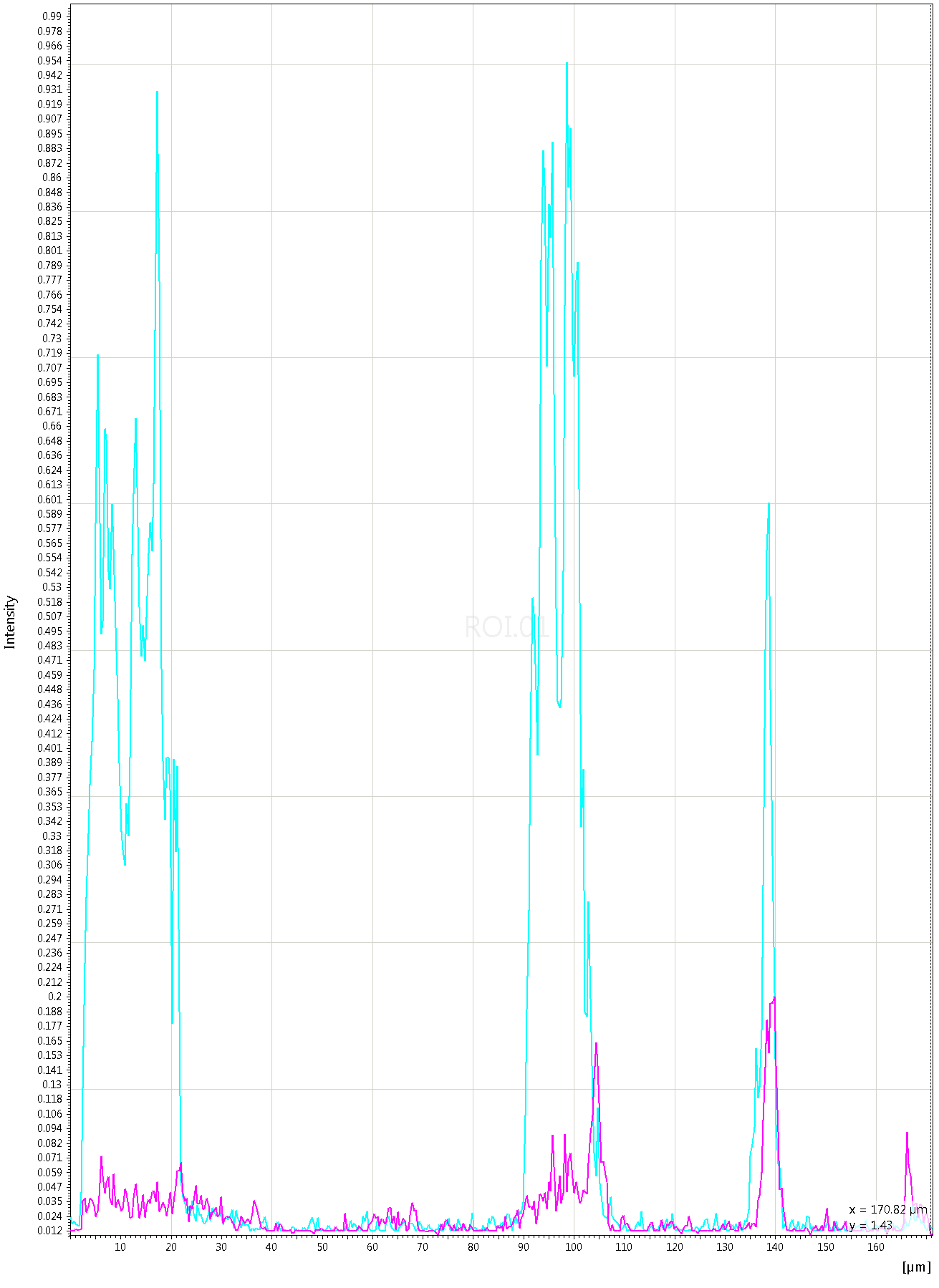

Supplement: S3 Fig — Cells were stained with anti-4G2 (pan-flavivirus antibody, pink fluorescence) and PAFI (DNA staining, blue fluorescence). Representative graph of fluorescence intensity (y-axis) against the distance in micrometer (x-axis) of infected PMA-treated THP-1 cells, obtained from confocal representative image (confocal microscope Leica TCS-SP8 at 200X magnification). n = 2. (TIF) [file pone.0267653.s003.tif]
